# Supplementary material for: PLATE-Seq for genome-wide regulatory network analysis of high-throughput screens
Source: Nat Commun. 2017 Jul 24;8:105. doi: 10.1038/s41467-017-00136-z (PMC5524642; doi:10.1038/s41467-017-00136-z)
Supplement: Supplementary file 1 — Supplementary Information [file 41467_2017_136_MOESM1_ESM.pdf]

File name: Supplementary Information
Description: Supplementary figures, supplementary tables and supplementary references.

File name: Peer review file
Description:

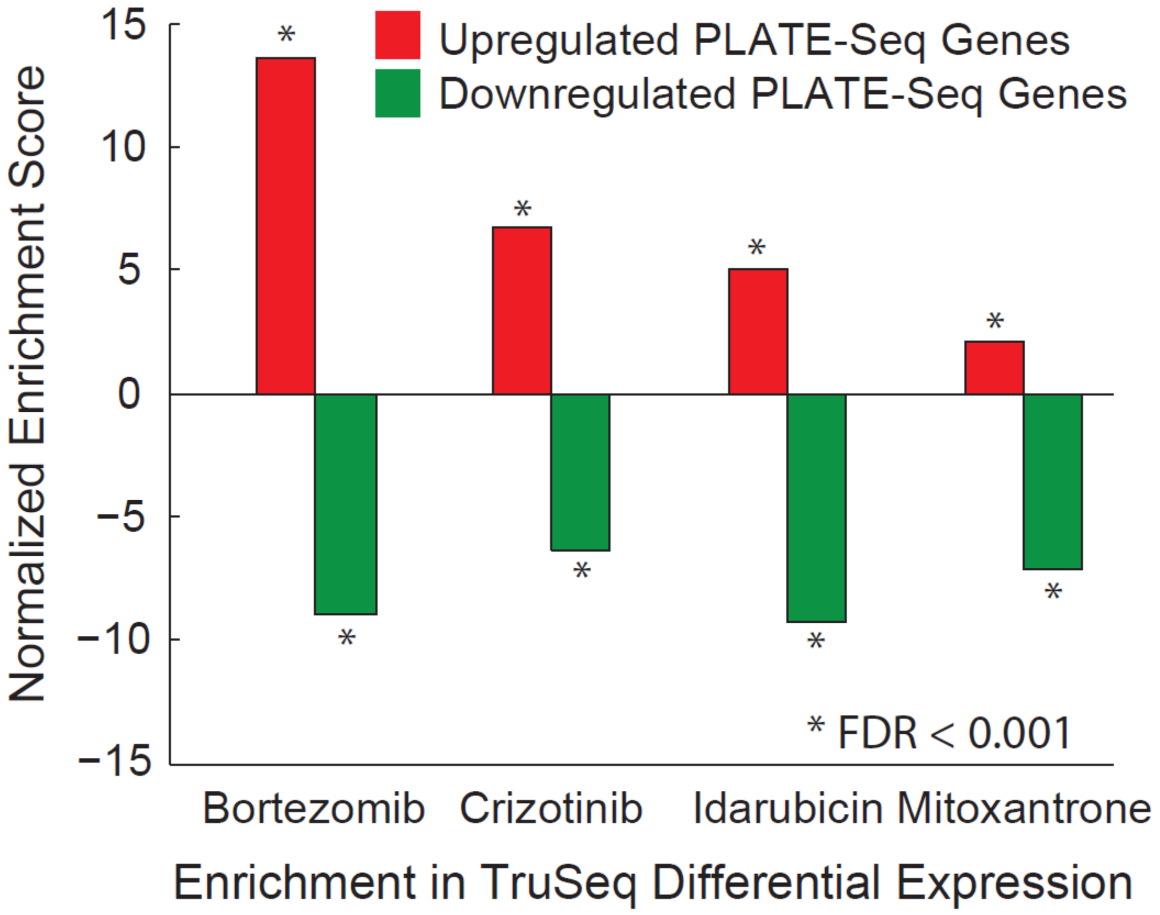

Supplementary Figure 1. Gene set enrichment analysis (GSEA) conducted using gene sets derived from the set of differentially upregulated or downregulated genes identified from DESeq2 analysis of the PLATE-Seq experiment from **Figure 2**. Enrichment and depletion of these gene sets was assessed on the same samples measured using conventional RNA-Seq (Illumina TruSeq) where genes were ranked by +/-log(q) where q is the FDR. This number is positive for upregulated and negative for downregulated genes. Pre-ranked, classical GSEA was conducted using the GSEA package from the Broad Institute[1]. FDR in the figure indicates the GSEA FDR. For each drug, the PLATE-Seq targets exhibited high-amplitude normalized enrichment scores in the appropriate direction.

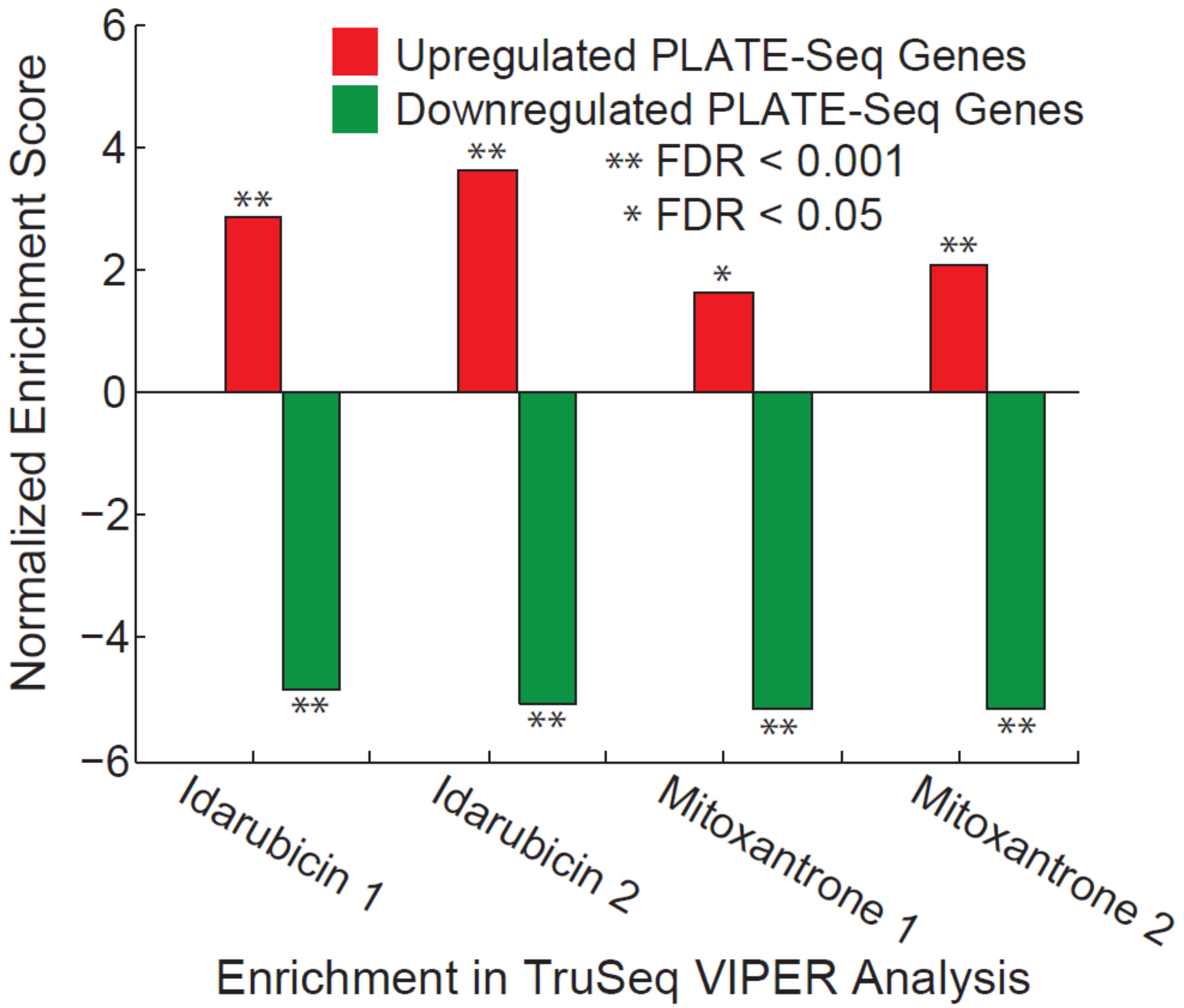

Supplementary Figure 2. Gene set enrichment analysis (GSEA) conducted using gene sets derived from the set of differentially activated or inactivated genes identified from VIPER analysis of the PLATE-Seq experiment from **Figure 2**. Enrichment and depletion of these gene sets was assessed on the same samples measured using conventional RNA-Seq (Illumina TruSeq) where genes were ranked by their VIPER NES. Pre-ranked, classical GSEA was conducted using the GSEA package from the Broad Institute[1]. FDR in the figure indicates the GSEA FDR. For each TruSeq sample, the PLATE-Seq targets exhibited high-amplitude normalized enrichment scores in the appropriate direction.

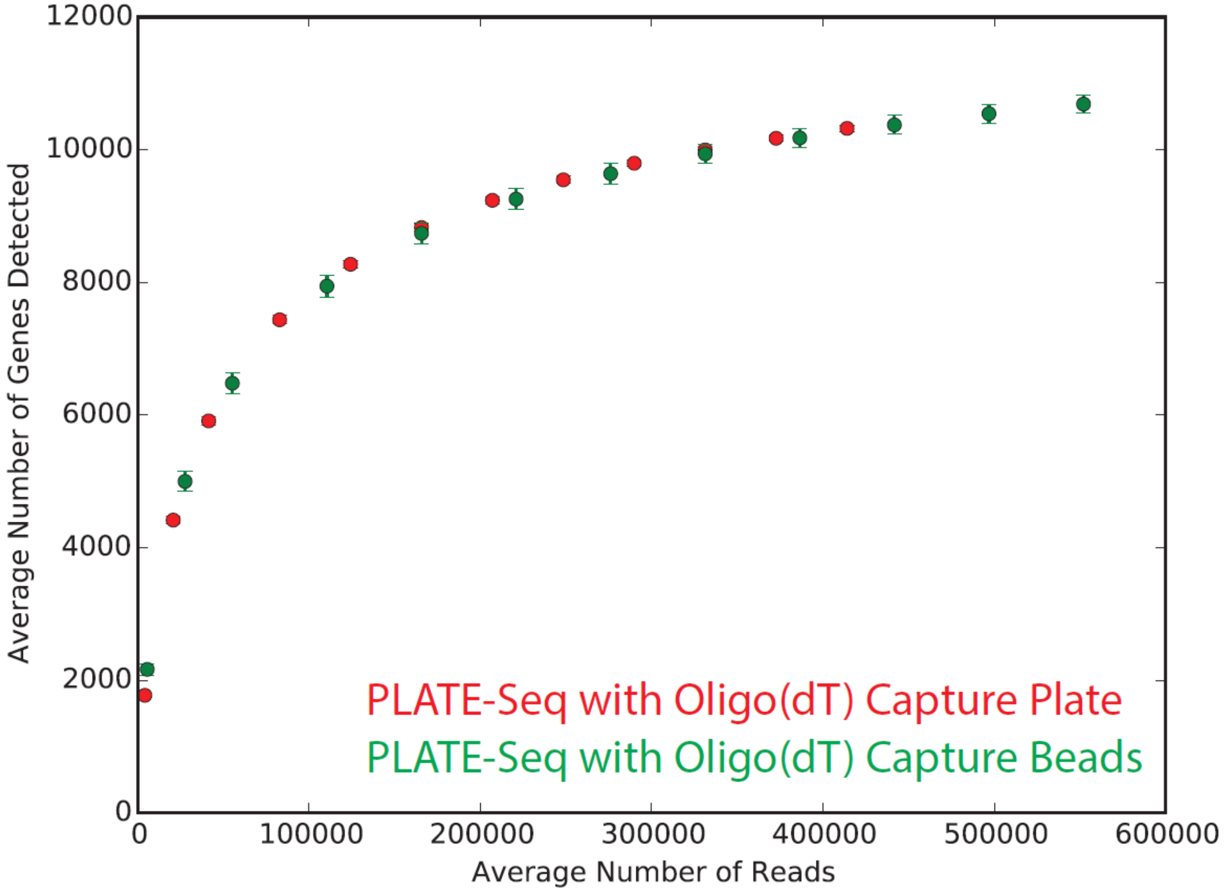

Supplementary Figure 3. Gene detection saturation curves for 96 PLATE-Seq samples obtained using conventional column- and oligo(dT) bead-based mRNA purification and oligo(dT) capture plate-based mRNA purification based on random subsampling. The points represent the average over all 96 wells and the error bars are s.e.m.

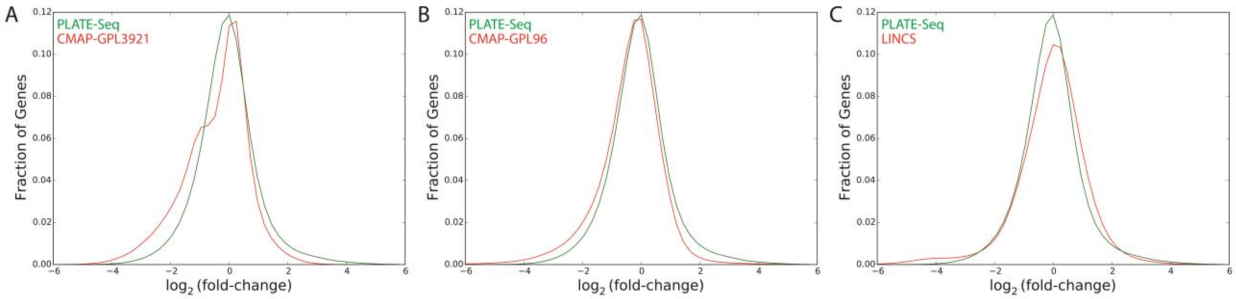

Supplementary Figure 4. Fold-change distributions for PLATE-Seq in comparison to A) CMap GPL3921 data set, B) CMap GPL96 data set, and C) LINCS data set for each non-zero expression measurement relative to the average over vehicle controls.

Supplementary Table 1. Table of drugs and concentrations uses in large-scale PLATE-Seq screen in U87 cells (**Figure 3**).

Drug	Concentration (uM)
Bortezomib	0.01
Fulvestrant	0.03
Mitoxanthrone dihydrochloride	0.01
Raloxifene	0.01
Ibrutinib	0.35
Topotecan hydrochloride	0.10
Mithramycin A	0.08
Trametinib (GSK1120212)	0.04
Ixazomib (MLN2238)	0.04
Panobinostat (LBH589)	0.01
Carfilzomib (PR-171)	0.00
Sunitinib Malate	0.05
Dasatinib	0.35
Dactinomycin	0.00
Busulfan	0.30
Bosutinib	0.38
Azacitidine	0.39
Arsenic trioxide	0.40
Epirubicin Hydrochloride	0.16
Ponatinib (AP24534)	0.14
Bleomycin sulfate	0.28
Tofacitinib citrate (CP-690550 citrate)	0.31
Axitinib	0.14
Octreotide in Water	0.01
Ixabepilone	0.00
Paclitaxel	0.01
Afatinib	0.19
Crizotinib	0.18
Gefitinib	0.57
Clofarabine	0.16
Cladribine	0.17
Vinorelbine	0.00
Cobimetinib (GDC-0973, RG7420)	0.50
Mercaptopurine	0.87
Doxorubicin	0.08
Vinblastine	0.01
Romidepsin	0.70
Gemcitabine	0.01
Thioguanine	0.59

Lenvatinib (E7080)	0.65
Ceritinib (LDK378)	0.75
Teniposide	0.63
Palbociclib (PD-0332991) HCl	0.10
Daunorubicin hydrochloride	0.07
Idarubicin	0.02
Pomalidomide	0.21
Decitabine	0.64
Temsirolimus	0.69
Cabazitaxel (Jevtana)	0.00
Leuprolide (Leuprorelin; Lupron)	0.08
Omacetaxine / Homoharringtonine	0.02
Toremifene	1.60
Belinostat	3.50
Etoposide	3.00
Bexarotene	1.60
Exemestane	1.50
Mitomycin C	1.90
Tamoxifen	0.96
Imatinib	5.50
Cytarabine	1.40
Sorafenib	4.90
Docetaxel	6.40
Tretinoin	2.10
Olaparib (AZD2281)	4.00
Vandetanib (ZD6474)	7.20
Mechlorethamine	0.88
Nilotinib (AMN-107)	2.29
Osimertinib (AZD9291)	1.60
Melphalan	0.82
Amsacrine	1.80
Erlotinib	3.40
Vorinostat	1.40
Carmustine	1.60
Oxaliplatin	1.60
Folinic acid calcium salt	2.10
Fludarabine	3.20
Lenalidomide	1.70
Regorafenib (BAY 73-4506)	5.20
Letrozole	1.90
Irinotecan	1.50
Dabrafenib (GSK2118436)	1.60
Vemurafenib (PLX4032)	6.50

Cisplatin	6.30
Sonidegib / Erismodegib	5.90
Cabozantinib - XL 184	1.10
Pentostatin	1.02
Alectinib (CH5424802)x	0.33
Hydroxyurea	10.00
Lapatinib Ditosylate (Tykerb)	10.00
Vismodegib (GDC-0449)	10.00
Mitotane	8.40
Pazopanib	8.60
OSI-906 (Linsitinib)	9.92
OTX015	3.91
P276-00	0.44
Pacritinib (SB1518)	0.57
PCI-24781	0.34
Perifosine (KRX-0401)	5.00
PF-04691502	0.15
Picoplatin	2.24
Pimasertib (AS-703026)	0.91
Pirarubicin	0.01
Pixantrone (BBR-2778), dimaleate salt	7.11
Plinabulin (NPI-2358)	1.57
Pracinostat (SB939)	0.42
Prinomastat hydrochloride	0.47
Propranolol HCl	1.62
PX 12	2.95
Quizartinib (AC220)	0.58
RAF265	4.94
Raltitrexed	0.01
Refametinib (RDEA119, Bay 86-9766)	2.29
Resminostat	4.44
RO4929097	1.10
Rocilinostat (ACY-1215)	1.54
Rucaparib (AG-014699, PF-01367338)	3.23
SAR245409 (XL765)	0.50
Satraplatin (JM216)	1.71
Savolitinib (Volitinib; AZD6094; HMPL-504)	11.40
SCIO 469 hydrochloride	4.81
Selinexor (KPT-330)	0.14
Sofrastaurin	11.33
TAK-700 (Orteronel)	11.71
TAK-733	0.27

Talampanel	1.14
Talazoparib (BMN673)	0.06
Tandutinib (MLN518)	1.02
Tariquidar	1.88
Tasisulam	20.00
Tasquinimod	0.52
Telatinib	2.35
Tirapazamine	20.00
Tivozanib (AV-951)	0.18
Triapine	5.48
Triciribine	0.78
UCN-01	0.07
Varlitinib	5.78
Vindesine sulphate	0.02
Volasertib (BI 6727)	1.33
Voreloxin (SNS-595)	1.23
XL147	20.00
Zibotentan (ZD4054)	1.66
SULCONAZOLE NITRATE	3.09
AMINACRINE	7.55
BENZALKONIUM CHLORIDE	7.82
ACRIFLAVINIUM HYDROCHLORIDE	8.45
CELASTROL	8.37
HOMIDIUM BROMIDE	3.26
PRISTIMERIN	0.86
4 -METHOXYCHALCONE	9.87
TETRACHLOROISOPHTHALONITRILE	3.94
DIHYDROCELASTROL	9.19
2,6-DIMETHOXYQUINONE	7.99
DIALLYL TRISULFIDE	0.88
SAPPANONE A DIMETHYL ETHER	2.36
7-DESACETOXY-6,7-DEHYDROGEDUNIN	2.00
TOTAROL	4.34
THYMOQUINONE	0.99
KINETIN RIBOSIDE	4.87
PLUMBAGIN	2.04
IPAG	8.92
Tetrindole mesylate	7.60
Methyl 2,5-dihydroxycinnamate	0.86
A-7 hydrochloride	7.87
3-CPMT	9.08
RS 17053 hydrochloride	2.91

WIN 64338 hydrochloride	8.51
(R)-(-)-Niguldipine hydrochloride	2.75
NSC 663284	7.79
SDM25N hydrochloride	8.47
ZK 93423	8.33
ER 27319 maleate	1.48
NNC 05-2090 hydrochloride	6.18
IKK 16	2.23
2,3-DCPE hydrochloride	3.53
Lylamine hydrochloride	1.98
10-DEBC hydrochloride	3.89
GW 843682X	1.05
Bax channel blocker	2.51
INCA-6	2.29
Ryuvidine	3.56
CGP 71683 hydrochloride	1.33
Nebivolol hydrochloride	2.74
LY 2183240	0.90

Supplementary Table 2. Table of primer sequences used in PLATE-Seq.

PLATEseq_second_strand	GCCTTGGCACCCGAGAATTCCANNNNNNN
PLATEseq_oligodTBC1	GTTCAGAGTTCTACAGTCCGACGATCTGAACACGTTTTTTTTTTTTTTTTTTTTTTTTT
PLATEseq_oligodTBC2	GTTCAGAGTTCTACAGTCCGACGATCCGTTGTCATTTTTTTTTTTTTTTTTTTTTTTTT
PLATEseq_oligodTBC3	GTTCAGAGTTCTACAGTCCGACGATCTGAATCAGTTTTTTTTTTTTTTTTTTTTTTTTT
PLATEseq_oligodTBC4	GTTCAGAGTTCTACAGTCCGACGATCTTCGACTGTTTTTTTTTTTTTTTTTTTTTTTTT
PLATEseq_oligodTBC5	GTTCAGAGTTCTACAGTCCGACGATCCTTAGTTGTTTTTTTTTTTTTTTTTTTTTTTTT
PLATEseq_oligodTBC6	GTTCAGAGTTCTACAGTCCGACGATCCGTGCTGATTTTTTTTTTTTTTTTTTTTTTTTT
PLATEseq_oligodTBC7	GTTCAGAGTTCTACAGTCCGACGATCCCTCAAGCTTTTTTTTTTTTTTTTTTTTTTTTT
PLATEseq_oligodTBC8	GTTCAGAGTTCTACAGTCCGACGATCTGAGGACTTTTTTTTTTTTTTTTTTTTTTTTTT
PLATEseq_oligodTBC9	GTTCAGAGTTCTACAGTCCGACGATCCGCGTTATTTTTTTTTTTTTTTTTTTTTTTTTT
PLATEseq_oligodTBC10	GTTCAGAGTTCTACAGTCCGACGATCCGTTTCATTTTTTTTTTTTTTTTTTTTTTTTTT
PLATEseq_oligodTBC11	GTTCAGAGTTCTACAGTCCGACGATCGCATAGTCTTTTTTTTTTTTTTTTTTTTTTTTT
PLATEseq_oligodTBC12	GTTCAGAGTTCTACAGTCCGACGATCTGGCTCTATTTTTTTTTTTTTTTTTTTTTTTTT
PLATEseq_oligodTBC13	GTTCAGAGTTCTACAGTCCGACGATCCAAGGAAGTTTTTTTTTTTTTTTTTTTTTTTTT
PLATEseq_oligodTBC14	GTTCAGAGTTCTACAGTCCGACGATCCAGTACCTTTTTTTTTTTTTTTTTTTTTTTTTT
PLATEseq_oligodTBC15	GTTCAGAGTTCTACAGTCCGACGATCCGACTTGTTTTTTTTTTTTTTTTTTTTTTTTTT
PLATEseq_oligodTBC16	GTTCAGAGTTCTACAGTCCGACGATCATGCCTCATTTTTTTTTTTTTTTTTTTTTTTTT
PLATEseq_oligodTBC17	GTTCAGAGTTCTACAGTCCGACGATCTCTCAGAATTTTTTTTTTTTTTTTTTTTTTTTT
PLATEseq_oligodTBC18	GTTCAGAGTTCTACAGTCCGACGATCCTTCGTCTTTTTTTTTTTTTTTTTTTTTTTTTT
PLATEseq_oligodTBC19	GTTCAGAGTTCTACAGTCCGACGATCTTCACATGTTTTTTTTTTTTTTTTTTTTTTTTT
PLATEseq_oligodTBC20	GTTCAGAGTTCTACAGTCCGACGATCAAAGCGAGTTTTTTTTTTTTTTTTTTTTTTTTT
PLATEseq_oligodTBC21	GTTCAGAGTTCTACAGTCCGACGATCCGCGAATCTTTTTTTTTTTTTTTTTTTTTTTTT
PLATEseq_oligodTBC22	GTTCAGAGTTCTACAGTCCGACGATCTAGAGATCTTTTTTTTTTTTTTTTTTTTTTTTT
PLATEseq_oligodTBC23	GTTCAGAGTTCTACAGTCCGACGATCCCCAAACATTTTTTTTTTTTTTTTTTTTTTTTT
PLATEseq_oligodTBC24	GTTCAGAGTTCTACAGTCCGACGATCTTGGAAACTTTTTTTTTTTTTTTTTTTTTTTTT
PLATEseq_oligodTBC25	GTTCAGAGTTCTACAGTCCGACGATCAGACTCGCTTTTTTTTTTTTTTTTTTTTTTTTT
PLATEseq_oligodTBC26	GTTCAGAGTTCTACAGTCCGACGATCTTTTACCGTTTTTTTTTTTTTTTTTTTTTTTTT
PLATEseq_oligodTBC27	GTTCAGAGTTCTACAGTCCGACGATCGCTAACGGTTTTTTTTTTTTTTTTTTTTTTTTT
PLATEseq_oligodTBC28	GTTCAGAGTTCTACAGTCCGACGATCCATAACCCTTTTTTTTTTTTTTTTTTTTTTTTT
PLATEseq_oligodTBC29	GTTCAGAGTTCTACAGTCCGACGATCTTCCCGCATTTTTTTTTTTTTTTTTTTTTTTTT
PLATEseq_oligodTBC30	GTTCAGAGTTCTACAGTCCGACGATCAACAGTTGTTTTTTTTTTTTTTTTTTTTTTTTT
PLATEseq_oligodTBC31	GTTCAGAGTTCTACAGTCCGACGATCTAGCTAGCTTTTTTTTTTTTTTTTTTTTTTTTT
PLATEseq_oligodTBC32	GTTCAGAGTTCTACAGTCCGACGATCGCAACTAGTTTTTTTTTTTTTTTTTTTTTTTTT
PLATEseq_oligodTBC33	GTTCAGAGTTCTACAGTCCGACGATCGGTTTCGCTTTTTTTTTTTTTTTTTTTTTTTTT
PLATEseq_oligodTBC34	GTTCAGAGTTCTACAGTCCGACGATCGAGTTACTTTTTTTTTTTTTTTTTTTTTTTTTT
PLATEseq_oligodTBC35	GTTCAGAGTTCTACAGTCCGACGATCTCCAAGTCTTTTTTTTTTTTTTTTTTTTTTTTT
PLATEseq_oligodTBC36	GTTCAGAGTTCTACAGTCCGACGATCTAGCATACTTTTTTTTTTTTTTTTTTTTTTTTT
PLATEseq_oligodTBC37	GTTCAGAGTTCTACAGTCCGACGATCATCTAGGATTTTTTTTTTTTTTTTTTTTTTTTT
PLATEseq_oligodTBC38	GTTCAGAGTTCTACAGTCCGACGATCATCATGTGTTTTTTTTTTTTTTTTTTTTTTTTT
PLATEseq_oligodTBC39	GTTCAGAGTTCTACAGTCCGACGATCTCTCTAGTTTTTTTTTTTTTTTTTTTTTTTTTT
PLATEseq_oligodTBC40	GTTCAGAGTTCTACAGTCCGACGATCAGGAAGAATTTTTTTTTTTTTTTTTTTTTTTTT

PLATEseq_oligodTBC41	GTTCAGAGTTCTACAGTCCGACGATCACGACCTCTTTTTTTTTTTTTTTTTTTTTTTTT
PLATEseq_oligodTBC42	GTTCAGAGTTCTACAGTCCGACGATCACACGCCATTTTTTTTTTTTTTTTTTTTTTTTT
PLATEseq_oligodTBC43	GTTCAGAGTTCTACAGTCCGACGATCGTCAAGGTTTTTTTTTTTTTTTTTTTTTTTTTT
PLATEseq_oligodTBC44	GTTCAGAGTTCTACAGTCCGACGATCCTCCCCTTTTTTTTTTTTTTTTTTTTTTTTTTT
PLATEseq_oligodTBC45	GTTCAGAGTTCTACAGTCCGACGATCCGCAAAATTTTTTTTTTTTTTTTTTTTTTTTTT
PLATEseq_oligodTBC46	GTTCAGAGTTCTACAGTCCGACGATCGTTAGCGTTTTTTTTTTTTTTTTTTTTTTTTTT
PLATEseq_oligodTBC47	GTTCAGAGTTCTACAGTCCGACGATCCCGGTGAATTTTTTTTTTTTTTTTTTTTTTTTT
PLATEseq_oligodTBC48	GTTCAGAGTTCTACAGTCCGACGATCATTGTCCTTTTTTTTTTTTTTTTTTTTTTTTTT
PLATEseq_oligodTBC49	GTTCAGAGTTCTACAGTCCGACGATCACTGACTTTTTTTTTTTTTTTTTTTTTTTTTTT
PLATEseq_oligodTBC50	GTTCAGAGTTCTACAGTCCGACGATCCGAGTAAATTTTTTTTTTTTTTTTTTTTTTTTT
PLATEseq_oligodTBC51	GTTCAGAGTTCTACAGTCCGACGATCTTACCGTGTTTTTTTTTTTTTTTTTTTTTTTTT
PLATEseq_oligodTBC52	GTTCAGAGTTCTACAGTCCGACGATCCCAGATACTTTTTTTTTTTTTTTTTTTTTTTTT
PLATEseq_oligodTBC53	GTTCAGAGTTCTACAGTCCGACGATCGGTGATCTTTTTTTTTTTTTTTTTTTTTTTTTT
PLATEseq_oligodTBC54	GTTCAGAGTTCTACAGTCCGACGATCTTTCTCCCTTTTTTTTTTTTTTTTTTTTTTTTT
PLATEseq_oligodTBC55	GTTCAGAGTTCTACAGTCCGACGATCGACTCCCATTTTTTTTTTTTTTTTTTTTTTTTT
PLATEseq_oligodTBC56	GTTCAGAGTTCTACAGTCCGACGATCCTTTCCCTTTTTTTTTTTTTTTTTTTTTTTTTT
PLATEseq_oligodTBC57	GTTCAGAGTTCTACAGTCCGACGATCCTACTGACTTTTTTTTTTTTTTTTTTTTTTTTT
PLATEseq_oligodTBC58	GTTCAGAGTTCTACAGTCCGACGATCGTGGACTTTTTTTTTTTTTTTTTTTTTTTTTTT
PLATEseq_oligodTBC59	GTTCAGAGTTCTACAGTCCGACGATCCTAGCATCTTTTTTTTTTTTTTTTTTTTTTTTT
PLATEseq_oligodTBC60	GTTCAGAGTTCTACAGTCCGACGATCTATCGGTCTTTTTTTTTTTTTTTTTTTTTTTTT
PLATEseq_oligodTBC61	GTTCAGAGTTCTACAGTCCGACGATCTGCAGCTGTTTTTTTTTTTTTTTTTTTTTTTTT
PLATEseq_oligodTBC62	GTTCAGAGTTCTACAGTCCGACGATCCAGGCGTATTTTTTTTTTTTTTTTTTTTTTTTT
PLATEseq_oligodTBC63	GTTCAGAGTTCTACAGTCCGACGATCTGCTTAACTTTTTTTTTTTTTTTTTTTTTTTTT
PLATEseq_oligodTBC64	GTTCAGAGTTCTACAGTCCGACGATCGTGTACACTTTTTTTTTTTTTTTTTTTTTTTTT
PLATEseq_oligodTBC65	GTTCAGAGTTCTACAGTCCGACGATCTTGCGGATTTTTTTTTTTTTTTTTTTTTTTTTT
PLATEseq_oligodTBC66	GTTCAGAGTTCTACAGTCCGACGATCTCATTGGATTTTTTTTTTTTTTTTTTTTTTTTT
PLATEseq_oligodTBC67	GTTCAGAGTTCTACAGTCCGACGATCGTACCCACTTTTTTTTTTTTTTTTTTTTTTTTT
PLATEseq_oligodTBC68	GTTCAGAGTTCTACAGTCCGACGATCGAATTGTGTTTTTTTTTTTTTTTTTTTTTTTTT
PLATEseq_oligodTBC69	GTTCAGAGTTCTACAGTCCGACGATCCAGTTCGGTTTTTTTTTTTTTTTTTTTTTTTTT
PLATEseq_oligodTBC70	GTTCAGAGTTCTACAGTCCGACGATCAGATGCTATTTTTTTTTTTTTTTTTTTTTTTTT
PLATEseq_oligodTBC71	GTTCAGAGTTCTACAGTCCGACGATCATTGTGGCTTTTTTTTTTTTTTTTTTTTTTTTT
PLATEseq_oligodTBC72	GTTCAGAGTTCTACAGTCCGACGATCATGTAACCTTTTTTTTTTTTTTTTTTTTTTTTT
PLATEseq_oligodTBC73	GTTCAGAGTTCTACAGTCCGACGATCCATGCCTTTTTTTTTTTTTTTTTTTTTTTTTTT
PLATEseq_oligodTBC74	GTTCAGAGTTCTACAGTCCGACGATCCCTCTTAGTTTTTTTTTTTTTTTTTTTTTTTTT
PLATEseq_oligodTBC75	GTTCAGAGTTCTACAGTCCGACGATCTACGAGCATTTTTTTTTTTTTTTTTTTTTTTTT
PLATEseq_oligodTBC76	GTTCAGAGTTCTACAGTCCGACGATCGGAAATTGTTTTTTTTTTTTTTTTTTTTTTTTT
PLATEseq_oligodTBC77	GTTCAGAGTTCTACAGTCCGACGATCGCAAGACGTTTTTTTTTTTTTTTTTTTTTTTTT
PLATEseq_oligodTBC78	GTTCAGAGTTCTACAGTCCGACGATCACTTTGCGTTTTTTTTTTTTTTTTTTTTTTTTT
PLATEseq_oligodTBC79	GTTCAGAGTTCTACAGTCCGACGATCGTGTGCTATTTTTTTTTTTTTTTTTTTTTTTTT
PLATEseq_oligodTBC80	GTTCAGAGTTCTACAGTCCGACGATCTGTTACGTTTTTTTTTTTTTTTTTTTTTTTTTT
PLATEseq_oligodTBC81	GTTCAGAGTTCTACAGTCCGACGATCCTGAGACGTTTTTTTTTTTTTTTTTTTTTTTTT
PLATEseq_oligodTBC82	GTTCAGAGTTCTACAGTCCGACGATCCCAGGCAATTTTTTTTTTTTTTTTTTTTTTTTT
PLATEseq_oligodTBC83	GTTCAGAGTTCTACAGTCCGACGATCGTCGCACATTTTTTTTTTTTTTTTTTTTTTTTT

PLATEseq_oligodTBC84	GTTCAGAGTTCTACAGTCCGACGATCGTCGACGATTTTTTTTTTTTTTTTTTTTTTTTT
PLATEseq_oligodTBC85	GTTCAGAGTTCTACAGTCCGACGATCGCCATTGGTTTTTTTTTTTTTTTTTTTTTTTTT
PLATEseq_oligodTBC86	GTTCAGAGTTCTACAGTCCGACGATCGAGACCGATTTTTTTTTTTTTTTTTTTTTTTTT
PLATEseq_oligodTBC87	GTTCAGAGTTCTACAGTCCGACGATCGCGCCTTATTTTTTTTTTTTTTTTTTTTTTTTT
PLATEseq_oligodTBC88	GTTCAGAGTTCTACAGTCCGACGATCTTCAGTCCTTTTTTTTTTTTTTTTTTTTTTTTT
PLATEseq_oligodTBC89	GTTCAGAGTTCTACAGTCCGACGATCCTTAGGCATTTTTTTTTTTTTTTTTTTTTTTTT
PLATEseq_oligodTBC90	GTTCAGAGTTCTACAGTCCGACGATCTCCTGTCATTTTTTTTTTTTTTTTTTTTTTTTT
PLATEseq_oligodTBC91	GTTCAGAGTTCTACAGTCCGACGATCAAGAGAGGTTTTTTTTTTTTTTTTTTTTTTTTT
PLATEseq_oligodTBC92	GTTCAGAGTTCTACAGTCCGACGATCTCGTTAAGTTTTTTTTTTTTTTTTTTTTTTTTT
PLATEseq_oligodTBC93	GTTCAGAGTTCTACAGTCCGACGATCTGTCCCCATTTTTTTTTTTTTTTTTTTTTTTTT
PLATEseq_oligodTBC94	GTTCAGAGTTCTACAGTCCGACGATCCACATTCCTTTTTTTTTTTTTTTTTTTTTTTTT
PLATEseq_oligodTBC95	GTTCAGAGTTCTACAGTCCGACGATCCGGTGATATTTTTTTTTTTTTTTTTTTTTTTTT
PLATEseq_oligodTBC96	GTTCAGAGTTCTACAGTCCGACGATCACGACTGTTTTTTTTTTTTTTTTTTTTTTTTTT

Supplementary References

1 Subramanian, A. *et al.* Gene set enrichment analysis: a knowledge-based approach for interpreting genome-wide expression profiles. *Proc Natl Acad Sci U S A* **102**, 15545-15550.
